# Methodological Insights into the Occurrence, Conversion, and Control of Polychlorinated Dibenzo-p-Dioxins/Dibenzofurans from Waste Incineration

**DOI:** 10.3390/molecules30204106

**Published:** 2025-10-16

**Authors:** Ruoru Xu, Xuetong Qu, Yunfei He, Feifei Chen, Yuchi Zhong, Hangjun Zhang, Jiafeng Ding, Jibo Dou

**Affiliations:** 1 Zhejiang Provincial Key Laboratory of Wetland Intelligent Monitoring and Ecological Restoration, School of Engineering, Hangzhou Normal University, Hangzhou 311121, China; 2 Hangzhou Fuyang Huilong Environmental Protection Technology Co., Ltd., Hangzhou 330183, China

**Keywords:** fly ash, dioxins, waste incineration, formation mechanisms, detoxification technologies, environmental risk

## Abstract

With the acceleration of global urbanization, polychlorinated dibenzo-p-dioxins/dibenzofurans (PCDD/Fs), as a by-product of solid waste incineration, have attracted more and more attention for their environmental pollution and health hazards. By describing the generation and transformation mechanism of PCDD/Fs, this review focuses on the methods to control the generation of PCDD/Fs and reduce their environmental pollution. Initially, the study analyzes the formation mechanisms of PCDD/Fs, and it emphasizes that variations in incineration conditions, feedstock compositions, and technological approaches substantially influence PCDD/F formation. Subsequently, the review examines existing PCDD/F control technologies—including optimization technology of high-temperature pyrolysis and incineration, photocatalytic degradation technology, supercritical water oxidation technology and biodegradation—and evaluates their respective advantages and limitations. The current challenges and future research directions, such as the development of novel monitoring technologies, the development of industry standards, and the enhancement of policy support, are finally presented. Effective PCDD/F control requires advanced real-time monitoring (e.g., AI-enhanced mass spectrometry), unified global standards, and policy support (e.g., subsidies, phased regulations). Future solutions lie in multiscale modeling, international collaboration, and adaptive technologies for sustainable risk reduction.

## 1. Introduction

**Figure 1:**
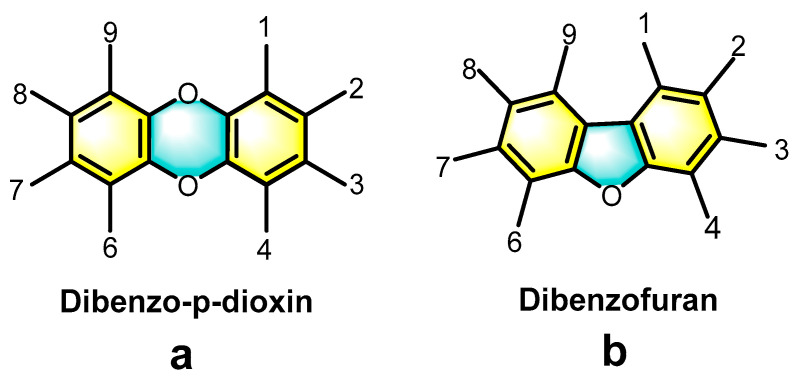
General structural formulas for (**a**) PCDDs and (**b**) PCDFs. Each numbered position can be substituted either by a hydrogen atom or by a chlorine atom (adapted from ref. [10]).

Over the last few decades, the globe has experienced significant urbanization and rapid economic growth, as reflected in the amount of municipal solid waste (MSW) generated by countries [1,2,3,4,5]. China’s MSW production, according to national annual data, has increased from 148 million tons in 2006 to 254 million tons in 2023 [6]. During this period, the proportion of waste incineration rose from 16% to 82%, with roughly 696 incineration plants built by 2023. Nevertheless, the emission of typical combustion byproducts—polychlorinated dibenzo-p-dioxins/dibenzofurans (PCDD/Fs)—has emerged as an inevitable challenge [7], hindering the global advancement of incineration technology in MSW management [8,9]. PCDD/Fs refer to a group of chlorinated aromatic compounds, which include polychlorinated dibenzo-p-dioxins (PCDDs) and polychlorinated dibenzofurans (PCDFs). PCDDs consist of two benzene rings connected by two oxygen atoms, as shown in Figure 1a. In contrast, PCDFs are composed of two benzene rings connected by an oxygen atom and a carbon–carbon bond, as illustrated in Figure 1b. The hydrogen atoms on the benzene rings can be replaced by chlorine atoms, resulting in 75 isomers of PCDDs and 135 isomers of PCDFs [10]. Moreover, they are highly stable in the environment, difficult to degrade, and prone to accumulating in the food chain, ultimately affecting the health of both humans and animals [11,12,13]. There is research that shows that these compounds are carcinogenic, and they are classified as Group I human carcinogens by the International Agency for Research on Cancer (IARC) [14]. In particular, 2,3,7,8-tetrachlorodibenzo-p-dioxin (2,3,7,8-TCDD) is considered to be one of the strongest known environmental carcinogens [15,16]. There is no doubt that these compounds are the most notorious class of persistent organic pollutants (POPs)—the Stockholm Convention prioritizes them as the “dirty dozens” [17,18].

The generation mechanism of PCDD/Fs during solid waste incineration is complex and involves mainly high-temperature gas-phase synthesis, precursor synthesis, and de novo synthesis [19,20]. The generation of PCDD/Fs mainly originates from incomplete combustion of chlorine-containing organics under high-temperature conditions [15]. In waste incineration treatment, chlorides and hydrocarbons generate dioxin-like substances through chemical condensation reactions [10]. In addition, organic precursors from incomplete combustion during waste incineration, such as polychlorinated benzenes (PCBs) and polychlorophenols (PCPs), and heterogeneous catalytic reactions in fly ash are also important pathways for the generation of PCDD/Fs [20,21,22]. Meanwhile brominated precursors can be converted to chlorinated products through electrophilic chlorination reactions, further increasing the proportion of site-specific substituted PCDD/F congeners [22,23,24]. The generation of PCDD/Fs during waste incineration is closely related to factors such as combustion conditions, raw material composition, and incineration technology. Therefore, a study of the formation mechanism of PCDD/Fs and the key factors affecting their generation is of great significance for the development of effective control and reduction strategies [25].

A variety of control technologies, such as activated carbon adsorption, catalytic reduction, and biological detoxification, have been proposed and applied for the effective control of PCDD/Fs [26,27,28,29,30]. These methods have their advantages and disadvantages and apply to different waste types and treatment conditions. However, these methods still face challenges in terms of economic issues, treatment efficiency, and environmental impacts [31,32], and new technologies and methods need to be developed to control and reduce the generation and emission of PCDD/Fs more effectively [33]. In addition, establishing industry standard evaluation technologies and obtaining strong support from national policies are all key to solving this problem. Through multifaceted efforts, the generation and emission of hazardous substances during waste incineration can be effectively reduced to protect the environment and human health.

Considering the importance of hazardous substance reduction during waste incineration and the complexity of the related processes, in this review, we aim to systematically investigate the recent advances in methodologies for controlling the generation of PCDD/Fs and reducing their environmental pollution in three aspects: (1) generation mechanisms and transformation processes, (2) control technologies, and (3) challenges and perspectives. First, we systematically summarize the latest research advances in methodologies for the generation, transformation, and control of PCDD/Fs during waste incineration, and then analyze the advantages and limitations of current technologies and propose potential solutions. Finally, we explore the challenges that hinder further reduction in PCDD/Fs and future directions for improving waste treatment technologies. This review aims to provide a scientific basis and policy recommendations to promote the sustainable development of waste treatment technologies.

## 2. PCDD/F Formation and Conversion

**Figure 2:**
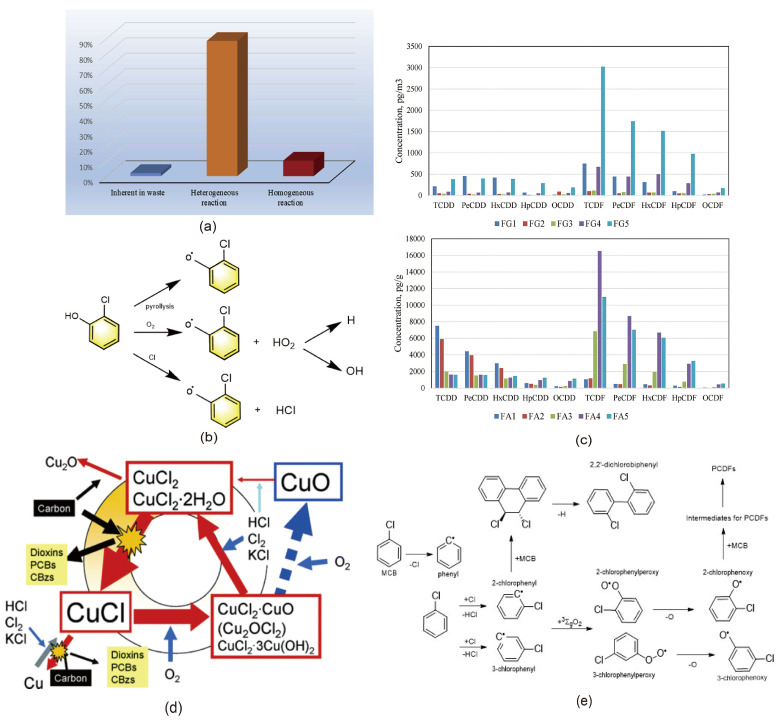
(**a**) The proportion of PCDD/Fs produced by different reactions to total PCDD/Fs in fly ash, reprinted with permission from ref. [34]. (**b**) Three formation modes of phenoxy radicals. (adapted from ref. [43]) (**c**) Behavior of copper in fly ash in the oxychlorination cycle, reprinted with permission from ref. [36]. (**d**) PCDD/F congener concentration distributions for flue gas and fly ash samples (FA stands for fly ash sample and FG stands for flue gas sample), reprinted with permission from ref. [55]. (**e**) Formation pathways of PCDFs from chlorobenzene (CB) precursor.

PCDD/F formation and conversion reactions have been a major focus of environmental pollution research for decades, with well-established theories that offer significant guidance for the rational control of dioxin emissions and the design of thermal technologies suited to different MSW sources. Consequently, it is necessary to start with the fundamentals of PCDD/F formation and conversion reactions, especially those occurring in MSW incineration plants. This is because the high dioxin content is mainly due to the large number of dioxins synthesized in the tail end of the incinerator during the operation of the large-scale incinerator, which is absorbed by the MSWI fly ash with a large surface area. One study showed the ratio of PCDD/Fs produced by different reactions, as shown in Figure 2a [34]. In this section, we clarify the mechanisms behind PCDD/F formation by presenting typical case studies and identifying the factors influencing these reactions. Additionally, we highlight innovative approaches to mechanistic investigations, spanning from molecular-level insights to macroscopic analyses.

### 2.1. Basic Principles of PCDD/F Formation During MSW Incineration

Since the discovery of dioxins in the flue gas and fly ash emitted from an Amsterdam waste incinerator by Olie in 1977 [35], researchers worldwide have conducted extensive studies on the mechanisms of dioxin formation during the combustion process [9,34,36]. The congener patterns of dioxin-like pollutants emitted during different combustion processes have been well-characterized in both experimental and theoretical investigations, and these outcomes have led to the conclusion that PCDD/Fs are mainly formed through three pathways:

Several studies proposed that dioxins are synthesized from monoaromatic compounds such as chlorophenol and chlorobenzene under pyrolytic or oxidative conditions in thermal systems, a process known as the “precursor synthesis pathway” [37,38]. Alternatively, in their “de novo” theory, Vog and Stieglitz highlighted that two elements—oxygen and metal—stand central in catalytic synthesis [39,40,41]. Another widely accepted regime is homogeneous synthesis, which proceeds in the gas phase at a higher temperature (>600 °C), in the absence of metal catalysis [42]. Thus, three pathways for the formation of dioxin-like compounds have been described.

#### 2.1.1. Homogeneous Gas-Phase Pathway

The homogeneous gas-phase synthesis pathway is one of the primary routes for the formation of dioxins during the initial stage of incineration [9]. This process mainly occurs in the high-temperature gas-phase environment of waste combustion. In this pathway, PCDD/Fs are formed through free radical reactions of halogenated organic compounds, such as chlorobenzene and chlorophenol, under high temperatures [43]. This process typically takes place in combustion zones where temperatures exceed 850 °C, involving the thermal decomposition of organic compounds and free radical recombination [44]. The core mechanism involves the thermal decomposition of chlorinated organic compounds at high temperatures, releasing chlorine radicals (Cl^•^), phenyl ring fragments, and oxygen radicals (e.g., ^•^OH). These free radicals undergo chain reactions and recombine to form polychlorinated aromatic intermediates, which ultimately condense to form PCDD/Fs. For example, chlorobenzene and chlorophenol undergo dechlorination reactions in high-temperature environments to generate phenoxyl radicals, which then couple to form PCDD/Fs [45]. Specifically, the high-temperature homogeneous gas-phase reactions primarily follow three steps: first, the generation of free radicals, where chlorobenzene and chlorophenol molecules thermally decompose to produce phenoxyl radicals (PhO^•^) and Cl^•^ [46], as shown in Figure 2b; second, free radical coupling, where the generated phenoxyl radicals couple with chlorine radicals or other phenoxyl radicals to form polychlorinated dibenzo-p-dioxin (PCDD) or polychlorinated dibenzofuran (PCDF) skeletons; and third, chlorination—that is, in the presence of chlorine (Cl_2_) or hydrochloric acid (HCl), the skeleton of PCDD or PCDF is further chlorinated to generate PCDD/Fs [21]. This process is greatly influenced by temperature and oxygen concentration. Too-high or too-low temperatures may affect the generation efficiency of PCDD/Fs, but high temperatures usually promote its generation [47]. Too much oxygen will lead to a complete oxidation reaction, while at a low oxygen concentration, the generation of PCDD/Fs will increase.

#### 2.1.2. De Novo Synthesis

De novo synthesis is another crucial pathway for the formation of PCDD/Fs. This process refers to the secondary low-temperature formation of PCDD/Fs on the surface of fly ash during the cooling stage of combustion flue gases [48,49,50]. In contrast to the homogeneous gas-phase pathway, de novo synthesis primarily occurs within the temperature range of 200 to 400 °C and involves interactions between carbonaceous materials, metal catalysts (such as CuCl_2_), and halogen sources [48,51]. During the de novo synthesis process, the carbonaceous materials in the fly ash serve as the reaction substrate, while metal oxides (e.g., copper, iron) act as catalysts, [52] facilitating the reaction between the carbonaceous materials and halogenated compounds, leading to the formation of PCDD/Fs [27,53]. The reaction proceeds through several steps: first, unburned carbon particles in the fly ash are oxidized by oxygen, generating phenoxyl radicals [43]. Subsequently, phenoxyl radicals undergo chlorination reactions in the presence of metal catalysts (e.g., Cu), producing polychlorinated dibenzo-p-dioxin (PCDD). Finally, the chlorinated compounds undergo further coupling reactions, resulting in the formation of PCDD/Fs [54]. Copper, iron, and other metals have a significant catalytic effect on the reaction of de novo synthesis. It is found that the amount of PCDD/Fs can be significantly increased by adding catalysts, such as CuCl_2_. Figure 2d shows the behavior of copper in the oxychlorination cycle of fly ash [54]. Initially, CuCl_2_ is reduced to CuCl through interaction with carbon, a process that simultaneously facilitates the chlorination of carbon to generate organochlorine compounds, including potentially hazardous substances such as dioxins. Subsequently, CuCl can be further reduced to elemental copper by carbon. The resulting CuCl is then oxidized by O_2_ to form copper oxychloride. Copper oxychloride reacts with Cl_2_ to regenerate the starting material, CuCl_2_, thereby completing the cycle. However, O_2_ has been proposed to disrupt this cycle by converting some intermediate compounds into copper oxides, thereby altering the reaction pathway and preventing the sustained operation of the cycle [36]. Future research should focus on the speciation of copper in fly ash, considering variables such as temperature, ash composition, and surrounding environmental conditions, as these factors are believed to influence the chemical forms of copper. Additionally, it is imperative to investigate the correlation between the formation of dioxins and the changes in copper speciation.

#### 2.1.3. Precursor Synthesis

Precursor synthesis is one of the key mechanisms for the formation of PCDD/Fs. Chen et al. (2018) found that the percentage of PCDD in FA1 (fly ash test group 1) and FA2 (fly ash test group 1) is much higher than that of PCDF, as shown in Figure 2c, indicating that precursor synthesis is the main way to form PCDD/Fs [55]. This process specifically refers to the generation of target pollutants through a series of reactions involving halogenated organic precursors during thermochemical conversion. This pathway typically occurs within the sub-high temperature range of 200–600 °C, with the most typical reaction temperature zone between 250 and 500 °C [56]. Its reaction kinetics significantly differ from those of high-temperature pyrolysis pathways. Typical precursor substances include chlorinated aromatic compounds, chlorinated phenolic compounds, and chlorinated naphthalene compounds, among others. These substances undergo complex multiphase reaction networks within the combustion system: first, they undergo thermal cleavage to generate active free radical intermediates, and then, under an oxidative atmosphere, the free radicals couple to form the dioxin skeleton structure [57]. This process is accompanied by continuous chlorination reactions, resulting in the directed substitution of chlorine atoms. Figure 2e shows the mechanism diagram of chlorobenzene (CB) oxidizing to form PCDFs. When the waste stream or upstream reaction produces more precursors (such as plastic additives or chlorophenol/chlorobenzene generated in the furnace), the precursor path and de novo synthesis share the catalytic/chlorination environment in the low-temperature section, resulting in synergy. In terms of rate, many experiments show that the unit reaction rate of the precursor path can be several orders of magnitude higher than that of alternative path, so its supply becomes the decisive factor [43]. Notably, this synthesis pathway is highly sensitive to the reaction micro-environment, and its efficiency is governed by a combination of multiple factors, including the degree of chlorination of the precursor, local oxygen concentration, and the catalytic effect of fly ash surfaces [58].

For the vast majority of MSWI under stable conditions, de novo synthesis is considered the dominant source; the contribution of precursor synthesis is second and strongly related to the content/formation of chlorinated aromatics in raw materials. The high-temperature gas phase is usually evaluated as a relatively small source. This judgment is based on the fact that the temperature range of 200–400 °C is called the “dioxin regeneration window”, which means that dioxins (PCDD/Fs) are most likely to be formed in this temperature range. This is because at this temperature, metal chlorides (such as chlorides of metals such as copper and iron) will help chemical reactions and promote the formation of dioxin-like precursors [3,36,59].

### 2.2. Factors That Affect the Yield of PCDD/F Products in MSW Incineration

The formation and transformation of PCDD/Fs result from the complex interaction and dynamic coupling of various environmental factors during waste incineration [60]. Key factors such as combustion temperature, residence time, oxygen levels, chlorine content, catalytic effects of metals, and fly ash characteristics all contribute to determining the pathways and emission profiles of PCDD/Fs. During MSW incineration, the generation and transformation of PCDD/Fs, which are significant pollutants, are strongly influenced by these factors, directly impacting their formation, emission characteristics, and toxicity. As environmental conditions change throughout the incineration process, the mechanisms driving PCDD/F formation also evolve. Thus, gaining a thorough understanding of how these environmental factors influence PCDD/F generation and transformation is crucial for optimizing the incineration process and controlling PCDD/F emissions.

#### 2.2.1. Combustion Temperature

Combustion temperature is a key factor in regulating the formation of PCDD/Fs. The results show that when the furnace temperature is lower than 600 °C, the path of precursors (such as chlorophenol and chlorobenzene) generating PCDD/Fs through an oxidative coupling reaction is dominant [61]. Studies have shown that within the 200–600 °C range, the oxidative coupling reactions of chlorinated precursors exhibit significant temperature dependence, with the reaction rate increasing exponentially with temperature [62]. In this temperature range, the pyrolysis of chlorinated organic compounds generates phenoxy radicals, which then undergo dimerization to form the dioxin backbone structure. Kinetic simulation results indicate that when the furnace temperature exceeds 850 °C with a residence time of ≥2 s, PCDD/Fs undergo efficient thermal decomposition, with their half-life at 1000 °C shortening to 0.1 s [63]. In the low-temperature zone of the flue gas duct (300–500 °C), metal catalysis facilitates the reformation of PCDD/Fs, with the formation rate peaking at approximately 350 °C [62]. To mitigate this process, rapid quenching technology can be employed to reduce the flue gas temperature to below 200 °C within one second. Optimization studies have demonstrated that maintaining the primary combustion zone temperature between 850 and 950 °C, combined with rapid cooling (T < 200 °C), not only significantly reduces the PCDD/F emission factor but also improves combustion efficiency [63].

#### 2.2.2. Oxygen Concentration

Oxygen concentration is another crucial environmental factor influencing the formation of PCDD/Fs during combustion [64]. The oxygen concentration in the combustion zone directly affects the oxidation degree of organic compounds and the formation of their byproducts, which in turn impacts PCDD/F generation. Higher oxygen concentrations promote the complete oxidation of precursors, thereby reducing the formation of PCDD/Fs due to incomplete combustion. For example, under excess oxygen conditions, chlorides in the waste are more easily oxidized, leading to a relatively lower yield of PCDD/Fs. In contrast, low oxygen levels lead to the pyrolysis of organic matter, generating more chlorinated aromatic free radicals, which serve as raw materials for subsequent coupling reactions. Under oxygen-deficient conditions, chlorobenzene, for instance, can undergo dechlorination to produce reactive chlorine atoms, which then combine with unburned carbon scaffolds to form highly chlorinated products [65]. Additionally, oxygen concentration also affects the formation pathways of PCDD/Fs. Studies indicate that under low-oxygen conditions, PCDD/Fs are primarily formed via de novo synthesis and precursor synthesis routes, whereas at high oxygen concentrations, the formation of PCDD/Fs is more likely to occur through homogeneous gas-phase reactions [66].

#### 2.2.3. Chlorine Sources

Chlorine sources play a crucial role in the formation of PCDD/Fs, primarily originating from chlorine-containing compounds found in waste materials, such as chlorinated plastics, chlorinated chemicals, and organic chlorinated compounds [27]. The type and concentration of these chlorine sources significantly influence the generation of PCDD/Fs during incineration. Research indicates that various types of chlorine sources contribute differently to PCDD/F formation. Organic chlorine compounds, such as PVC plastics and chlorophenols, release reactive chlorine during pyrolysis, and the incineration of these substances tends to significantly increase PCDD/F production [67]. On the other hand, inorganic chlorine compounds like NaCl need to undergo gas–solid phase reactions and involve fly ash catalysis to participate effectively in the formation process [68]. Furthermore, the structural properties of precursors also play an important role in the generation of PCDD/Fs. For example, 2,4,6-trichlorophenol, with chlorine atoms substituted at the neighboring positions, tends to form the highly toxic 2,3,7,8-TCDD more readily. This is because the lower spatial hindrance facilitates a lower activation energy for the carbon–oxygen coupling reaction [69]. Additionally, the concentration of chlorine sources directly determines the efficiency of PCDD/F generation. The greater the concentration of chlorine sources, the higher the amount of PCDD/Fs produced [67].

#### 2.2.4. Fly Ash Surface Conditions

The surface properties of fly ash play a crucial catalytic role in the generation and transformation of PCDD/Fs. The presence of metal catalysts, carbon content, and surface characteristics in fly ash significantly influence PCDD/F formation. Metals such as copper, iron, and nickel in fly ash can accelerate de novo synthesis of PCDD/Fs. For instance, CuCl_2_ has been identified as a key catalyst in PCDD/F generation. The catalytic mechanisms of these metals include redox cycles, surface adsorption activation, and synergistic effects [70]. Additionally, the carbon content in fly ash impacts PCDD/F production. Higher carbon concentrations generally promote PCDD/F formation, as carbon particles serve as reaction carriers, enhancing PCDD/F synthesis [71]. It is important to emphasize that the particle size and specific surface area of fly ash directly influence catalytic efficiency. Micron-sized fly ash particles (<10 μm) exhibit 2–3 times greater catalytic efficiency in PCDD/F generation compared to coarser particles (>50 μm), owing to their higher surface-active site density [72].

### 2.3. Strategies for Investigating PCDD/F Occurrence Processes

#### 2.3.1. Laboratory Simulations

**Figure 3:**
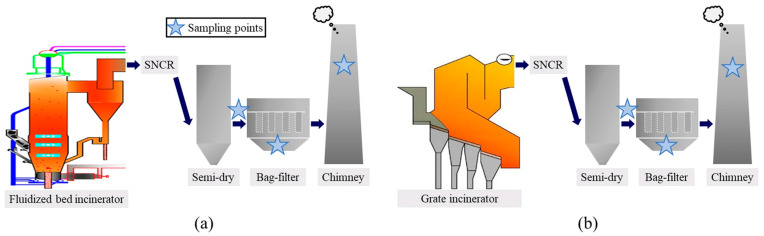
Schematic diagram of sample points for the fluidized bed (**a**) and grate incinerators (**b**). Reprinted with permission from ref. [78].

Laboratory simulations primarily replicate the incineration process by controlling temperature, residence time, oxygen concentration, and other factors to study how these conditions influence PCDD/F formation and distribution [28,56,73]. For example, the study on pyrolysis and oxidation of chlorobenzene, chlorinated aromatic hydrocarbons, and phenolic precursors at high temperatures is helpful for clarifying the formation path of PCDD/Fs, which helps elucidate the generation pathways of PCDD/Fs [74,75]. The role of metal catalysts, such as copper and iron, in PCDD/F formation is also explored to understand catalytic effects [76]. A common method for these simulations is using an incinerator to study chlorinated precursor conversion at specific temperatures, as shown in Figure 3. Samples containing chlorine sources and carbon sources are incinerated at 300–800 °C. Results show PCDD/F generation peaks around 600 °C, correlating with the highest rate of oxidative coupling reactions involving halogenated organics [27]. By controlling conditions, researchers analyze how each factor impacts PCDD/F formation in a controlled environment. Further progress can be made using isotopic labeling to trace halogenated aromatics’ coupling pathways or by introducing fly ash to study its catalytic influence on PCDD/F generation [77]. In addition, there are several pairs of typical experimental simulation devices, such as those shown in Table 1.

**Table 1:** Comparison between parameters of typical experimental simulation devices and applicable scenarios.

Device Type	Temperature Range (°C)	Gas Environment Control	Applicable Research Objectives
Tubular Furnace System [79]	20–1200	Static/dynamic atmosphere	Analysis of precursor pyrolysis path and determination of kinetic parameters.
Fixed-Bed Reactor [80]	100–800	Adjustable O_2_/N_2_/Ar flow	Study catalytic effects of metal oxides (e.g.,Cu/Fe) on PCDD/F formation via surface reactions.
Fluidized-Bed Reactor [81]	200–900	Turbulent mixing simulation	Study on catalytic effect of fly ash and the gas–solid mass transfer process.
Plasma Reactor [82]	20–2000	High-energy plasma (Ar/O_2_/H_2_O)	Explore ultra-fast decomposition pathways of PCDD/Fs under extreme energy conditions.
Fly Ash Interface Simulator [83]	20–1000	Surface chemical modification	Characterization of catalytic activity of metal–carbon interface.
Rotary Kiln Simulator [84,85]	800–1600	Continuous gas flow with variable O_2_	Simulate industrial-scale combustion conditions to analyze ash residues and thermal degradation.

#### 2.3.2. On-Site Inspection and Online Monitoring Technology

**Figure 4:**
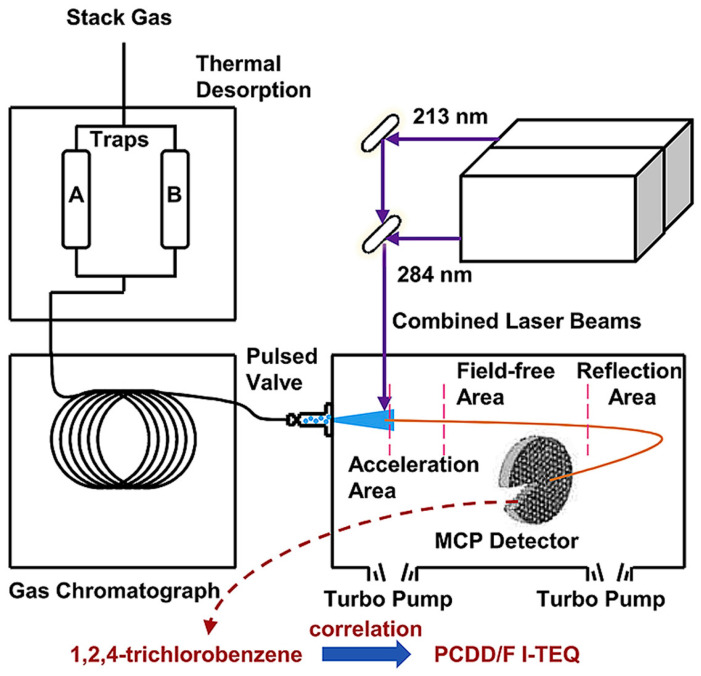
Schematic of TD-GC-REMPI-TOF-MS. (Reprinted with permission from ref. [96]).

During waste incineration, on-site monitoring plays a critical role in the real-time tracking of PCDD/F emissions. High-resolution gas chromatography coupled with high-resolution mass spectrometry (HRGC/HRMS) has been widely used in the detection of PCDD/Fs due to its high-precision quantitative analysis capabilities [86,87,88]. This technology offers exceptionally high sensitivity, enabling the detection of PCDD/F congeners at concentrations as low as pg/g [55]. When coupled with an online HRGC-HRMS system in a fluidized bed incinerator, it becomes possible to continuously monitor fluctuations in PCDD/F concentrations at various time points throughout the incineration process, thereby capturing the dynamic formation and transformation processes of these compounds in real-time [89]. However, the HRGC/HRMS technique is limited by its complex operational procedures and time-consuming sample preparation, which restricts its application in real-time monitoring. Recent advancements have led to the development of online monitoring methods based on resonance-enhanced multiphoton ionization time-of-flight mass spectrometry (REMPI-TOF-MS). Cao et al. developed thermal desorption (TD) gas chromatography and resonance-enhanced multiphoton ionization TOF-MS (TD-GC-REMPI-TOF-MS, Figure 4) for quantifying 1,2,4-trichlorobenzene and predicting the concentrations of PCDD/Fs in the stack gas of a municipal solid waste incinerator (400 t day^−1^). This system integrates TD, gas chromatography (GC), and REMPI-TOF-MS for highly selective analysis of dioxin compounds. The diagram illustrates the workflow from thermal desorption to mass spectrometry analysis. These methods, along with other technologies, help address these limitations [90]. These methods allow for the real-time monitoring of precursor substances such as chlorobenzene and chlorophenols, enabling indirect inference of PCDD/F formation trends through the changes in these precursors. This technique enables the real-time monitoring of variations in key PCDD/F precursors, such as chlorinated volatile organic compounds, offering valuable insights into the formation pathways of PCDD/Fs. By monitoring and analyzing these precursors, a more comprehensive understanding of the formation and transformation mechanisms of PCDD/Fs during waste incineration can be attained, providing theoretical support for the effective control of their emissions. Each of the key analytical techniques used to study the formation and transformation mechanisms of PCDD/Fs has its distinct advantages and limitations, as shown in Table 2.

#### 2.3.3. Molecular Dynamics Simulation and Quantum Chemical Calculation

**Figure 5:**
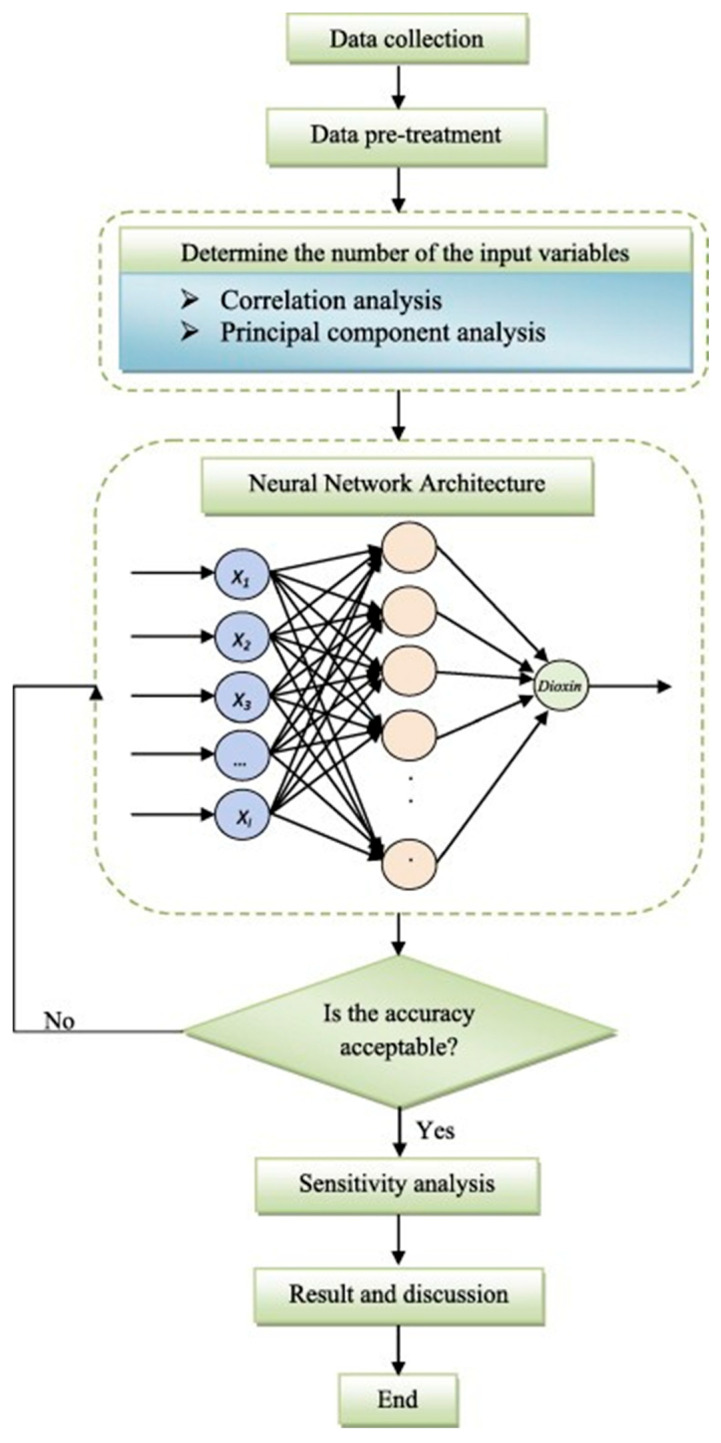
Flowchart of the artificial neural network approach for predicting the dioxin emission. Reprinted with permission from ref. [104].

Theoretical models are also key tools for exploring the microscopic mechanisms of PCDD/F formation. Among these models, quantum chemical calculations based on density functional theory (DFT) can predict the coupling pathways and energy barriers of chlorinated aromatic compounds. For example, in chlorophenols, the transition state energy for the C-O coupling reaction is lower due to the relatively smaller steric hindrance caused by ortho-substitution, which is consistent with the ortho-selective reaction behavior observed in experiments [97]. Molecular dynamics simulations, on the other hand, provide detailed insights at the atomic level into the specific process of CuO catalyzing the dehydrogenation of chlorophenols on the surface of fly ash [98]. A related study used molecular dynamics (MD) simulations to describe the atomic-level details of the interaction between chlorophenol molecules and the CuO surface, finding that the reaction rate is influenced by the surface defects of the catalyst and the adsorption patterns of the reactants. This simulation not only helps us understand the specific steps of the reaction but also reveals the impact of temperature and reaction conditions on the reaction mechanism [99,100]. The multiphase reaction network model integrates multiple processes, including gas-phase radical reactions, fly ash surface catalysis, and particulate adsorption, to predict the flux of PCDD/F generation. Simulations have found that within the temperature range of 300–500 °C in the flue gas duct, fly ash-mediated “secondary synthesis” can contribute 40–60% of the total emissions [101]. Additionally, machine learning optimization has emerged as an important tool for studying the PCDD/F formation mechanism [102,103]. By training neural network models with combustion parameters such as temperature, oxygen concentration, and waste composition, dynamic predictions of emission factors can be made, with errors effectively controlled within ±15% [104]. Figure 5 shows a flow chart of an artificial neural network method for predicting dioxin emission. These theoretical models provide a comprehensive analysis of the microscopic mechanisms of dioxin generation, offering a solid theoretical foundation and effective technical methods for further research and control of dioxin production [104].

## 3. Current Technologies for PCDD/Fs Detoxification

In the process of environmental pollution control of PCDD/Fs, detoxification technology plays a crucial role. Currently, researchers have developed a variety of technological tools for the degradation and transformation of PCDD/Fs, covering physical, chemical, and biological control strategies, etc. The high-temperature pyrolysis and incineration optimization technology destroys the chemical structure of PCDD/Fs through high-temperature conditions; the photocatalytic degradation technology utilizes light energy-driven redox reactions to convert PCDD/Fs into low-toxicity or non-toxic substances [105,106,107]; the supercritical water oxidation technology realizes the complete oxidative decomposition of PCDD/Fs in a supercritical water environment; and the biological detoxification technology is mainly realized through the metabolic activities of microorganisms biotransformation of PCDD/Fs, mainly through microbial metabolic activities [108]. Each of these technologies has its characteristics in terms of principle, application scope, advantages, and disadvantages, which will be introduced in detail next.

### 3.1. Optimization Technology of High-Temperature Pyrolysis and Incineration

High-temperature pyrolysis integrated with incineration optimization represents an approach employed extensively in waste-to-energy facilities and industrial boilers for the efficient decomposition and elimination of hazardous substances like PCDD/Fs from waste materials [109]. It has been proven that the emission concentration of PCDD/Fs can be significantly reduced by installing an incineration optimization system combined with tail rapid cooling technology. For example, a fluidized-bed incinerator project implemented the “secondary combustion chamber + SNCR denitrification” process, successfully lowering the PCDD/F concentration in flue gas to below 0.05 ng TEQ/Nm^3^ (TEQ, Toxicity Equivalency Quotient, is an index used to assess the toxicity of various chlorinated dibenzo-p-dioxins (PCDD/Fs) and dioxin-like compounds) [110,111]. The fundamental principle of this technology lies in the destruction of the chemical structure of PCDD/Fs. This is accomplished through high-temperature cracking and oxidation reactions [112], which are achieved by accurately controlling parameters such as temperature, reaction time, turbulence levels, and the amount of excess air during the incineration process.

**Figure 6:**
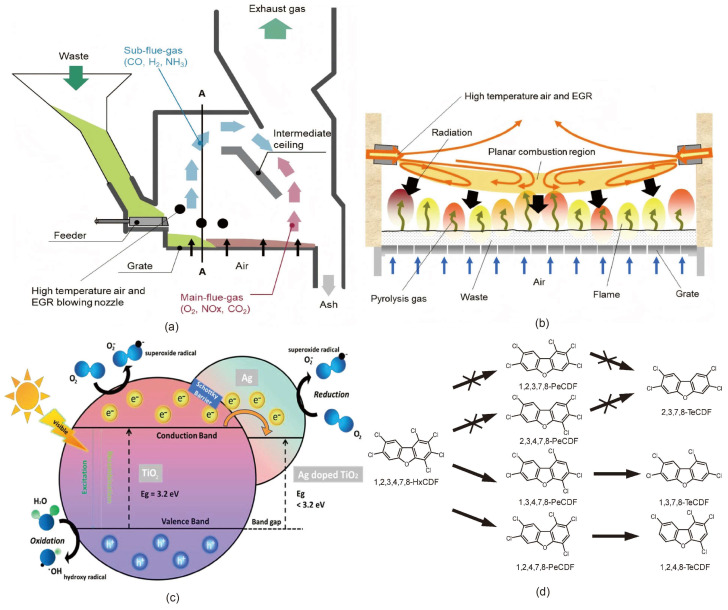
(**a**) Two-way gas flow incinerator based on HiCOT. Reprinted with permission from ref. [114]. (**b**) Conceptual rendering of the widthwise cross-section of the furnace inside based on the side-wall-blowing method. Reprinted with permission from ref. [114]. (**c**) Illustrates the process of charge transfer in Ag-doped TiO_2_ photocatalyst in photocatalytic degradation. Reprinted with permission from ref. [107]. (**d**) Pathways of the dechlorination of 1,2,3,4,7,8-HxCDF by a mixed culture containing *D. ethenogenes* strain 195. Reprinted with permission from ref. [115].

The specific mechanisms are detailed as follows: (1) High-temperature decomposition: Under elevated temperatures, PCDD/Fs undergo thermal decomposition, breaking the C-Cl bonds in their molecular structure. This process yields smaller, less toxic compounds. Studies indicate that when incineration temperatures reach or exceed 850 °C with a residence time of at least 2 s, the decomposition efficiency of PCDD/Fs can surpass 99.9% [113]. (2) Rapid cooling technology: During the flue gas cooling phase, the implementation of rapid cooling techniques—such as reducing the temperature to below 200 °C within 1 s—can effectively suppress secondary synthesis reactions [59]. These reactions, often catalyzed by metals like copper (Cu) and iron (Fe) present on fly ash surfaces, are responsible for the reformation of PCDD/Fs at lower temperatures. (3) Secondary combustion chamber design and high-temperature air combustion technology (HiCOT): Incorporating a secondary combustion chamber within the incinerator, operating at temperatures exceeding 1000 °C, and extending the residence time of flue gases enhance the removal efficiency of PCDD/Fs [111]. In addition, using HiCOT can realize stable low-air-ratio combustion, ensure more uniform combustion, and promote the secondary combustion process. Figure 6a and Figure 6b, respectively, illustrate furnaces utilizing high-temperature mixed gases and blowing recycled exhaust gas from the ceiling [114]. This approach helps prevent the formation of PCDD/F precursors, such as chlorobenzene and chlorophenol.

Despite its efficiency in decomposing organic matter, this method entails notable drawbacks and challenges. For instance, Cu/CuCl_2_ present in fly ash strongly catalyzes de novo synthesis of PCDD/Fs at 250–400 °C. Mitigation strategies include the judicious introduction of sulfur- or phosphorus-containing additives (e.g., SO_2_, phosphates) to convert active copper sites into inactive phases (such as copper sulfide and copper phosphate), increase the S/Cl ratio, diminish the effective chlorination capacity, and—operationally—limit copper input through feedstock control [115]. Although anoxic pyrolysis generally suppresses PCDD/F formation, it can generate precursors (e.g., chlorophenols and chlorobenzenes) that readily convert to dioxins under favorable conditions. Operational transients—such as start-ups, shutdowns, and load fluctuations—can further perturb residence time and temperature profiles, undermining the coordination between secondary combustion and rapid quenching. Current research therefore prioritizes intensifying secondary-air mixing and ensuring sufficient high-temperature residence time [116]. In addition, there is a problem of fly ash treatment—the fly ash, after rapid cooling, may be rich in heavy metals and dioxin precursors. At present, efficient fly ash solidification or melting treatment technology is adopted to solve this problem [117].

### 3.2. Supercritical Water Oxidation (SCWO) Technology: Impact of Oxidants on PCDD/F Degradation

**Figure 7:**
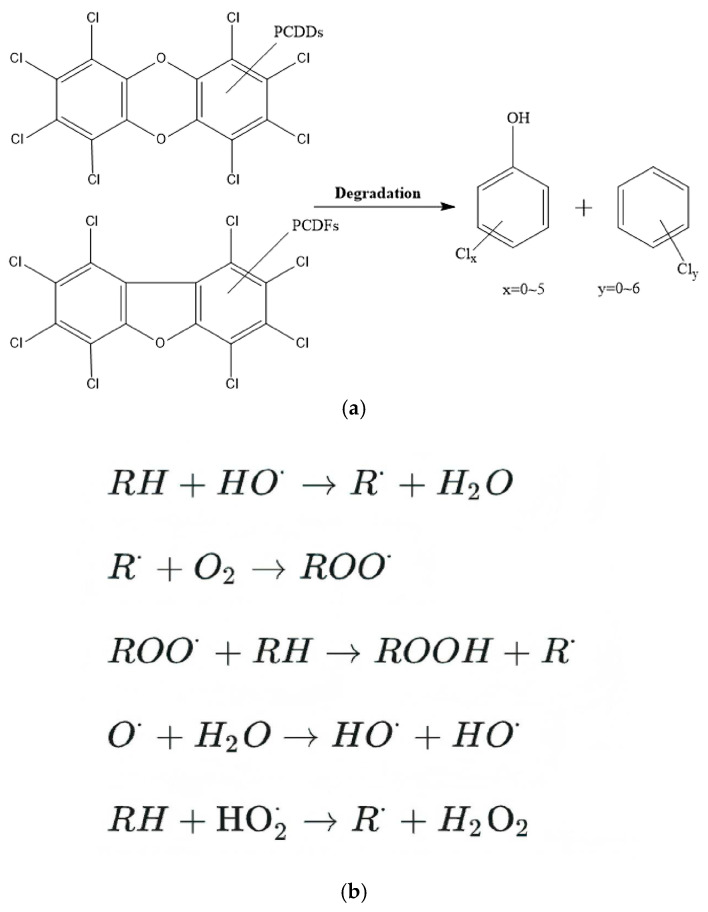
Decomposition processes of PCDD/Fs. The C-O bond in the molecular structure of dioxins (**a**) breaks down to form chlorobenzene or chlorophenol (which have a single benzene ring) and is further degraded by free radical oxidation (**b**). Reprinted with permission from ref. [34].

SCWO is a technology that utilizes the properties of water in a supercritical state to completely oxidize and decompose organic pollutants into harmless substances. In the supercritical state, water exhibits unique physicochemical properties [118], such as reduced dielectric constant and surface tension, high diffusion coefficient, and complete mutual solubility with organic matter and oxygen, thus providing an ideal environment for the efficient degradation of PCDD/Fs [119]. In this environment, oxidizing agents such as oxygen or hydrogen peroxide can undergo homogeneous free radical chain reactions with PCDD/F molecules, converting them to CO_2_, H_2_O, and inorganic halides (e.g., HCl, HF) [120], without producing secondary pollutants. Experimental studies have shown that SCWO technology can completely oxidize and decompose PCDD/Fs in a short period of time, and the dechlorination efficiency of perchlorinated dibenzo-p-dioxins can reach more than 99% [44]. The degradation pathway is mainly C-O bond breaking to generate low-chlorinated derivatives, as shown in Figure 7, followed by ^•^OH attacking the benzene ring to form quinone intermediates, and further oxidation of the intermediates to small molecule acids to finally generate CO_2_ and H_2_O [34].

In related studies, the optimization of key parameters has had a significant impact on the reaction effect and process [121]. As far as temperature and pressure parameters are concerned, the PCDD/F degradation rate constant shows an exponential increase when the temperature exceeds 400 °C. However, once the temperature exceeds 600 °C, corrosion problems will occur in the equipment. In terms of the choice of oxidant type, the use of H_2_O_2_ as an oxidant produces 3–5 times more ^•^OH compared to O_2_ [122], providing a significant advantage for the detoxification of highly toxic pollutants. SCWO technology has the advantages of complete removal of pollutants, no secondary pollution, and energy self-sustainability. However, problems such as equipment corrosion, salting-out, and high energy consumption due to high temperature and pressure still need to be addressed [123]. Researchers are developing materials with higher corrosion resistance, such as titanium alloy and special ceramics, to prolong equipment service life. Meanwhile, the design of permeable-wall reactors (TWRs) helps reduce corrosion and salt deposition by forming a protective film on the inner wall of the reactor [124]. Furthermore, the use of catalysts and auxiliary fuels has been observed to enhance both the reaction rate and degradation efficiency. Quantum chemistry and molecular dynamics simulations provide valuable insights into reaction mechanisms and product distributions, offering a theoretical foundation for reactor design and the optimization of operational parameters [125].

### 3.3. Photocatalytic Degradation Technology

Photocatalytic degradation technology is a green chemical method based on the light energy-driven conversion of PCDD/Fs into low-toxicity or non-toxic small molecular compounds through the generation of reactive oxidizing species, such as hydroxyl radicals and superoxide anions [126], utilizing photocatalysts under light conditions [127]. This technology has been widely used in air purification, water treatment, and waste treatment due to its high efficiency, environmental friendliness, and sustainability. The photocatalytic degradation process mainly consists of four key steps, as shown in Table 3.

The selection of a suitable photocatalyst is crucial for degradation efficiency. The main photocatalysts available today are metal oxide catalysts, non-metallic semiconductor catalysts, and composite catalysts [130,131,132]. Titanium dioxide (TiO_2_) is one of the most classical photocatalysts, with the advantages of high chemical stability and low cost [133,134,135]; its forbidden bandwidth limits the response to visible light, but the light absorption range can be extended by doping with metal or composite graphene, etc. Ramesh et al. (2024) demonstrated that the incorporation of silver significantly extends the photocatalytic activity of TiO_2_ into the visible light range. This enhancement is attributed to the formation of a Schottky barrier, which suppresses the recombination of photogenerated electron–hole pairs, thereby improving photocatalytic efficiency [136], as shown in Figure 6c. Similarly, Suhaimi et al. (2025) reported that nitrogen doping effectively reduces the bandgap energy of TiO_2_, leading to improved visible light absorption [137]. The combination of graphene oxide (GO) with TiO_2_ was found to enhance the separation efficiency of photogenerated charge carriers through synergistic interactions, ultimately resulting in superior overall photocatalytic degradation performance [138].

The efficiency of photocatalytic degradation of PCDD/Fs is affected by several key factors. Goetz et al. (2009) found that UV light has an effect on the degradation rate of pollutants, and developed a mathematical model that can be applied to the design of the reactor and scale-up production [139]. Catalyst concentration may also affect the degradation effect, and a too-high catalyst concentration may lead to light scattering and reduce the light utilization efficiency. The photolysis efficiency of the catalyst also varies under different organic solvents, and Choi et al. (2004) found that the photolysis rate of OCDD was related to the specificity of the solvent [140]. The pH of the solution significantly influences the surface properties and reaction rate of the catalyst. It has been demonstrated that TiO_2_ achieves optimal photocatalytic activity under neutral or mildly acidic conditions. The concentration of oxygen in the solution affects the redox process of the photocatalytic reaction, and the separation efficiency of photogenerated carriers decreases in an environment with a low oxygen concentration, leading to a decrease in the photocatalytic degradation efficiency [141]. Therefore, ensuring sufficient oxygen supply in the reaction system is an important condition to improve the photocatalytic performance of TiO_2_. At the same time, this technology may have to face catalyst deactivation. For example, halogen ions can inhibit the key steps of catalytic reaction, such as preventing the formation of holes (+) and hydroxyl radicals (OH), which are important elements for decomposing pollutants, and finally lead to the reduction in photocatalytic reaction efficiency. Moreover, when photocatalysis occurs in an environment containing complex substances, the deposition of organic substances and the scaling of inorganic salts (such as calcium salts and magnesium salts) will cover the surface of the catalyst, which will hinder the contact between light and reactants, resulting in the catalyst losing its effectiveness. Scientists are studying how to modify the catalyst surface to reduce the interference of chloride ions, optimize the reaction conditions to make the catalyst more durable, and use photoelectric catalysis technology to improve the stability and anti-interference ability of the catalyst [142]. In addition, when PCDD/Fs or their precursors undergo photocatalysis, partial dechlorination or rearrangement is often observed, leading to the formation of lower-chlorinated homologues or other chlorinated by-products. In some cases, photo-treatment of precursors (such as triclosan and chlorophenol) can even result in the generation of PCDD/Fs. To address this issue, current research trends focus on distinguishing between homologues and assessing toxicity using the Toxicity Equivalency Quotient (TEQ), with an emphasis on employing combined enzyme-linked immunosorbent assay (ELISA)/gas chromatography–mass spectrometry (GC-MS) characterization techniques. Additionally, efforts are being made to achieve deep mineralization (i.e., ring cleavage to CO_2_/H_2_O) in materials and processes, including the selection of appropriate wavelengths and composite catalysts to minimize residual toxicity [143].

### 3.4. Biological Detoxification Technology

Despite the high effectiveness of traditional physical and chemical degradation methods, they pose significant second pollution, high energy consumption, and complex facilities. In contrast, the biological detoxification method, characterized by its low cost, sustainability, and ease of operation, has gained extensive attention and application [144,145,146]. The biodegradation approach leverages the metabolic activities of microorganisms or enzymes to convert PCDD/Fs into non-harmful substances. Research has shown that certain microorganisms, such as white-rot fungi and *Pseudomonas aeruginosa*, can degrade dioxins. White-rot fungi secrete lignin peroxidase (LiP) and manganese peroxidase (MnP), which facilitate the hydroxylation and cleavage of the aromatic rings in PCDD/Fs [147].

The microbial degradation mechanisms of dioxins mainly consist of oxidative degradation, dechlorination degradation, ring-opening degradation, and enzyme-catalyzed degradation. Oxidative degradation involves breaking down the aromatic ring structure of dioxins through oxidative reactions to generate harmless substances [148,149]. For example, *Rhodococcus* sp. *SAO101*, under the catalytic action of angular dioxygenase, can cleave the oxygen bond in dioxin molecules, initiating an oxidative ring-opening reaction and the subsequent formation of dihydroxy compounds [150]. Under anaerobic conditions, dechlorination degradation becomes the primary pathway for microbial degradation of dioxins. Studies indicate that anaerobic microorganisms can remove chlorine atoms from the dioxin molecule, thereby reducing its toxicity. The dechlorination pathway is illustrated in Figure 6d. It is important to note that dechlorination can be accomplished by removing lateral, peripheral, and transverse chlorine from the dioxin structure [151,152,153], and the dechlorination pathway may vary according to the microbial species and environmental conditions [152]. PCDD/Fs can also be degraded by leveraging the metabolic capabilities of plants. Hyperaccumulator plants, such as mustard, can activate PCDD/Fs in the soil through the secretion of organic acids from their root systems. When combined with the microbial–plant symbiotic system, this can reduce the concentration of PCDD/Fs in contaminated soils by 50–70%.

The efficiency of microbial degradation of dioxins is influenced by multiple factors, including the type and quantity of microorganisms, temperature, oxygen concentration, pH, pollutant concentration, and the presence of co-metabolites [154,155]. Table 4 shows some key influencing factors and optimization strategies for biodegradable PCDD/Fs. All these factors impact microbial activity and degradation efficiency. Although microbial degradation offers the advantages of environmental friendliness and low cost, its application still has some limitations. At the Caffaro contaminated site in Brescia, Italy, researchers conducted an 18-month greenhouse experiment to evaluate the remediation efficacy of different plants against PCDD/Fs. Results showed that the herbaceous plant *Festuca arundinacea* reduced soil PCDD/F concentrations by approximately 11% to 24% during remediation, with corresponding half-lives ranging from 2.5 to 5.8 years. This result indicates that the biodegradation process is often slow and requires a long time to yield significant results [156]. Ishii et al. (2009) investigated the degradation capacity of the fungus *Paecilomyces boydii* toward 2,3,7,8-TCDD. Experiments demonstrated that during the logarithmic growth phase, *P. boydii* could degrade 2,3,7,8-TCDD using glucose as a carbon source, exhibiting a certain degradation capacity. However, this degradation rate was relatively slow and constrained by environmental conditions and the growth state of the microorganisms [157]. Additionally, new harmful intermediates may be produced during the degradation process [158], and some microorganisms may only be capable of degrading low-chlorinated dioxins, with limited degradation ability for high-chlorinated dioxins [159]. Current research focuses on using anaerobic reductive dehalogenating bacteria to first reduce and dechlorinate high-chlorinated PCDD/Fs into more readily oxidizable low-chlorinated forms, followed by aerobic bacteria performing *dioxygenase* ring-opening to achieve mineralization [148]. In addition to the traditional anaerobic dehalogenating bacterium *Dehalococcoides mccartyi*, recent studies have, for the first time, linked the reduction and dechlorination of 2,3,7,8-TCDD to the genus *Dehalobium*, suggesting the potential for developing new dehalogenase libraries and habitat sources [160]. In conclusion, through optimizing environmental conditions, screening high-efficiency strains, and modifying microbial metabolic pathways, it is anticipated that degradation efficiency can be enhanced, and the existing technical bottlenecks can be overcome.

By introducing the control technologies for the four PCDD/Fs, it can be observed that each possesses distinct advantages and disadvantages, resulting in varying suitability conditions. Table 5 presents a comprehensive comparison of the four control technologies.

## 4. Challenges and Perspectives

While progress has been made in the development of detoxification technologies and control strategies for PCDD/Fs, significant challenges remain in their practical application. To more effectively mitigate the environmental risks posed by PCDD/Fs, future research and practice should focus on the following areas: the development of advanced monitoring technologies, the establishment of more robust industry standards, and the implementation of supportive national policies. These measures are crucial for advancing PCDD/F pollution control technologies and reducing their potential threat to both ecosystems and human health. This chapter will address these challenges and explore future directions from these four key perspectives.

### 4.1. Development of New Investigation Technologies

The generation and transformation of PCDD/Fs during waste incineration present significant environmental and health risks [168]. While progress has been made in understanding the mechanisms of PCDD/F generation and control methods, challenges persist in the integrated assessment of their removal mechanism and environmental risks [31,169]. Current monitoring techniques for PCDD/Fs depend on complex and costly laboratory analyses, such as small-scale incinerator experiments and other laboratory simulations [170]. Although these methods provide some insights into PCDD/F generation patterns, the disparity between laboratory conditions and real incineration environments can introduce bias in the results. In real-world settings, simplified models fail to accurately predict the dynamic evolution of multiphase reaction networks. Therefore, there is an urgent need to develop new techniques for the real-time monitoring and assessment of PCDD/Fs.

**Figure 8:**
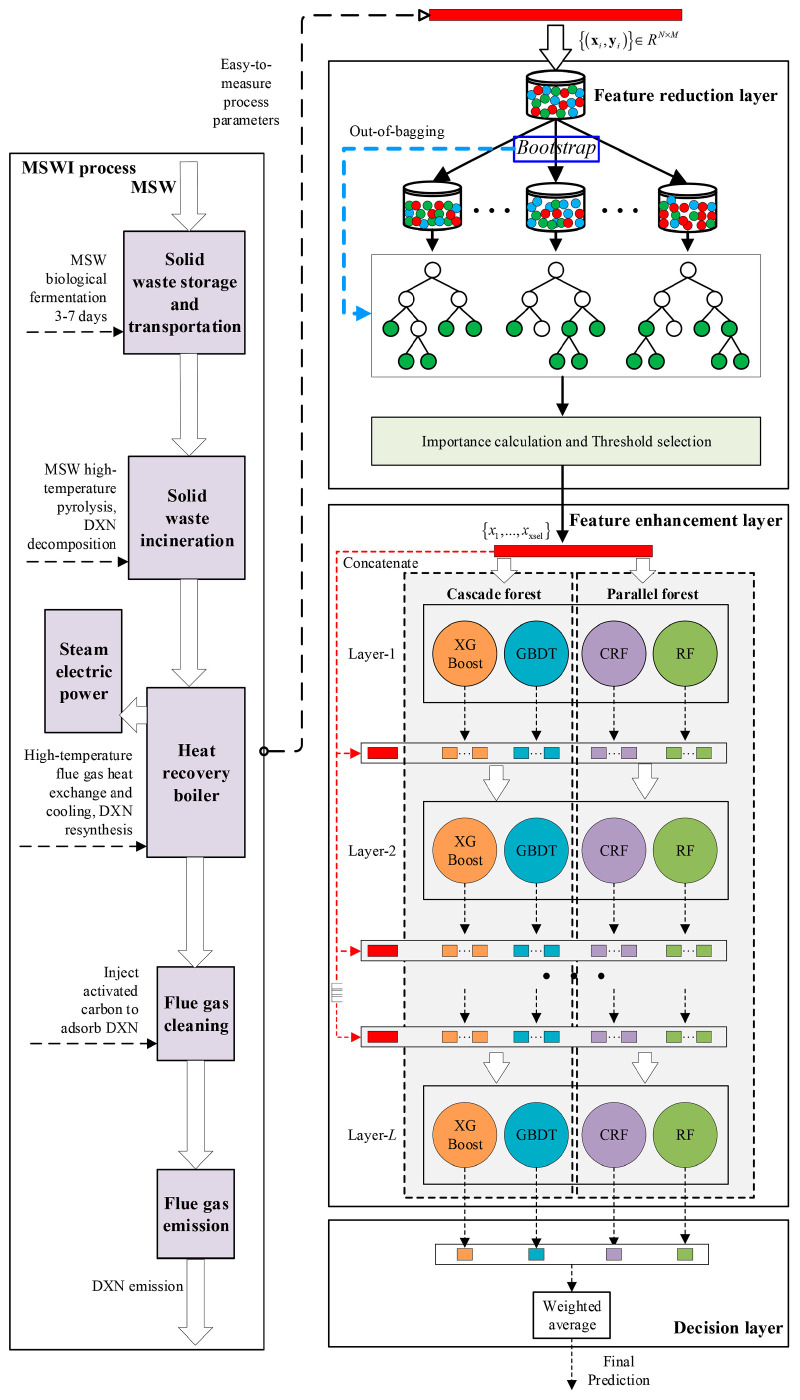
Diagram of ImDFR modeling strategy for prediction of dioxin emissions in MSWI processes. Reprinted with permission from ref. [174].

In recent years, researchers have attempted to indirectly estimate PCDD/F emission levels by correlating them with precursor substances such as chlorobenzene. For instance, Cai et al. proposed an online real-time monitoring method based on precursor substance correlations, enabling cost-effective real-time control of PCDD/Fs [171]. However, the accuracy and universality of these methods still require further validation. AI algorithms, particularly ensemble learning and deep learning methods, have been employed to predict the spatiotemporal variation in PCDD/Fs. For instance, Hsu et al. proposed an ensemble mixed spatial model (EMSM) based on geospatial data and AI to estimate daily average concentration variations in PCDD/Fs in Taiwan, achieving an explanatory power of 87% [172]. However, existing models are often based on specific regions or conditions, lacking adaptability across regions and time. Furthermore, the interpretability and transparency of these models require further improvement to gain broader acceptance in practical applications [172]. Additionally, low-temperature thermal treatment technology shows promising potential for addressing PCDD/Fs in municipal solid waste fly ash. Research indicates that within an appropriate temperature range (300–600 °C), this technology can effectively degrade PCDD/Fs, meeting the requirements of certain application scenarios [27]. However, such techniques still suffer from the problem of complex matrix interference. To address these problems, future research can focus on the following innovative directions: on the one hand, the development of highly sensitive detection techniques, such as optimization of the substrate surface morphology and electromagnetic field distribution. On the other hand, by analyzing and modeling a large amount of monitoring data [173,174], combining the catalytic effect of heavy metals and flue gas components with the dynamic parameter library generated by PCDD/Fs, and establishing a multifactor-coupled prediction model through machine learning algorithms, the efficiency and accuracy of monitoring can be improved [104,174,175]. A study used improved deep forest regression (ImDFR) to model dioxin emissions, and Figure 8 shows the overall strategy of dioxin emission prediction based on ImDFR.

In conclusion, in the future, we need to integrate various advanced detection techniques, build multiscale models, and utilize intelligent regulation systems to construct a risk assessment framework for PCDD/Fs.

### 4.2. Establishment of Industry Standards

The control of PCDD/Fs generated during waste incineration hinges on the establishment of unified industry standards. Currently, inconsistent monitoring methodologies and a lack of standardized protocols have led to significant discrepancies in reported PCDD/F emissions [33]. To address this, harmonized standards must be formulated, encompassing precise monitoring techniques, standardized operational parameters, and uniform emission limits. Furthermore, such standards should remain adaptable to integrate emerging technologies and evolving scientific insights.

To standardize the monitoring of PCDD/Fs, it is essential to validate existing monitoring methods to ensure their accuracy, specificity, and sensitivity [176]. The generation of PCDD/Fs is closely related to the incineration conditions, and therefore, key process parameters need to be specified through standards. Low-temperature cracking incineration technology, through the integration of multidisciplinary advantages, achieves the pre-decomposition of organic matter at the low-temperature stage, followed by high-temperature incineration to destroy the structure of the PCDD/Fs. Technological parameters, such as the cracking temperature gradient and the gas residence time, need to be clearly defined according to the standard. Currently, emission limits for dioxin-like substances are not standardized worldwide. For instance, the European Union standard sets a limit of 0.1 ng TEQ/Nm^3^ for dioxins in waste incineration flue gases, while some developing countries still adhere to more lenient standards [177]. The formulation of industry standards should be based on risk assessment modeling and progressively tightened by regional environmental conditions. For example, China could consider adopting the European Union’s approach, gradually reducing the limit from 0.5 ng TEQ/Nm^3^ to 0.1 ng TEQ/Nm^3^, supported by technical specifications for real-time monitoring [177]. China GB 18485-2014 “Emission Control Standards for Municipal Solid Waste Incineration” [178] specifies an emission limit of 0.1 ng I-TEQ/Nm^3^ for flue gas (under standard conditions). In China, following implementation of GB 18485-2014, PCDD/F concentrations in stack gas from multiple MSW incineration plants have generally fallen below the 0.1 ng I-TEQ/Nm^3^ standard. As documented in the literature, emission levels from MSWIs at nine incineration facilities ranged from approximately 0.016 to 0.29 ng I-TEQ/Nm^3^, with only a few medical waste incinerators (HWIs) exceeding the standard [179].

We face multiple challenges in establishing industry standards. First, PCDD/Fs have a complex chemical structure with many isomers, which makes it difficult to standardize analytical methods. Second, the concentration and composition of PCDD/Fs produced by different industries vary greatly, which requires the development of differentiated standards for specific industries. In addition, there are differences in analytical equipment and technology across different countries and regions. For example, some developing countries may lack advanced analytical equipment to meet the requirements of international standards [180]. In the future, with the continuous progress of analytical technology and the strengthening of international cooperation, the industry standards for PCDD/Fs are expected to be gradually unified. In conclusion, the establishment of industry standards for PCDD/Fs is a complex and critical process that necessitates global collaboration. Through the continuous advancement of analytical methods and the promotion of standardization, we can more effectively address the environmental challenges posed by PCDD/Fs.

### 4.3. Strong Support from National Policy

National policies play a crucial role in controlling the generation and emission of PCDD/Fs. Governments have promoted the development and application of PCDD/Fs abatement technologies through the enactment of stringent environmental regulations [181]. For example, the EU has mandated BAT permits to ensure compliance with the 0.1 ng I-TEQ/Nm^3^ standard, and the EU requires member states to implement BAT-associated emission levels in permits. For the incineration sector, it continues and refines stack-level controls for PCDD/Fs, complemented by process combinations including continuous monitoring and activated carbon injection/dry scrubbing. China has achieved alignment with international standards through mandatory national standards and a regulatory system, while the United States uses New Source Performance Standard/Emissions Guidelines to cover multiple categories of incineration sources and quantifies emissions to TEQ equivalents, providing a clear path for engineering compliance. However, the improvement of the policy framework also needs to take into account regional characteristics. For example, developing countries, due to differences in technological levels and economic capacity, can formulate a phased implementation plan, prioritizing the promotion of low-cost and efficient technologies (e.g., low-temperature cracking incineration) and then gradually transitioning to a high-standard pollutant control system. In addition, the government should provide financial support and tax incentives to encourage enterprises and research institutions to carry out research, development, and application of PCDD/F emission reduction technologies. For example, our government encourages enterprises to adopt high-efficiency incineration technology through the waste incineration power generation tariff subsidy policy and, at the same time, establishes a special fund to support the research and development of dioxin abatement technology, which is used to support the research and demonstration projects of PCDD/F abatement technology [182]. These policies not only help reduce the environmental risks of PCDD/Fs but also promote the innovation and development of related technologies, providing a strong guarantee for the realization of sustainable development. The control of PCDD/Fs is a global issue that requires the joint efforts of the international community. The government should actively participate in international organizations, such as the United Nations Environment Programme (UNEP), to share research results and technical experience and promote the development and implementation of global standards.

In summary, the support of national policies plays a key role in the popularization and application of control technologies for PCDD/Fs. By making strict laws and regulations, providing financial and technical support, and promoting international cooperation, the generation and environmental impact of PCDD/Fs can be effectively reduced, and the ecological environment and human health can be protected.

## Figures and Tables

**Table 2:** Performance comparison of key analysis technologies for PCDD/Fs.

Technical Name	Detectability	Resolution Ratio	Analysis Time	Application Area	Limitations
HRGC/HRMS [91]	pg/g level	Homologous separation	6–8 h	Quantitative analysis of total homologues.	Pretreatment is complicated and time-consuming.
TD-GC×GC-TOFMS [92]	ng/L to pg/g level	Two-dimensional separation	4–6 h	Identification of trace homolog in a complex matrix.	The instrument cost is high, and the operating threshold is high.
REMPI-TOFMS [93]	Trace substances ranging from ppt to ppb can be detected	Time resolution	Real-time monitoring	Online detection of precursors (such as chlorobenzene and chlorophenol).	Unable to directly detect PCDD/Fs, relying on indirect inference.
FTIR [94]	Percent level (functional group); ppm level (specific compound)	Functional group recognition	Real-time monitoring	Trend analysis of key precursors (HCl, CVOCs).	Low sensitivity, unable to distinguish homologues.
XANES [95]	ppm level	Chemical state resolution	A few minutes–1 h	Valence analysis of heavy metals in fly ash.	Needs a synchrotron light source.

**Table 3:** Key steps and mechanism of action in photocatalytic degradation of PCDD/Fs.

Step	Core Process	Key Influencing Factors	Ref.
Photoexcitation	Photon energy excites valence band electrons to jump to the conduction band, forming electron–hole pairs.	Light source wavelength (needs to be ≤387 nm for TiO_2_)	[128]
Electric charge migration	Electrons and holes migrate to the surface of the catalyst and participate in the reduction (to -O_2_-) and oxidation (to -OH) reactions, respectively.	Catalyst surface defects and crystalline phase structure.	[129]
Reactive oxygen attack (ROA)	-OH and -O_2_- attack the C-Cl bond, triggering dechlorination and aromatic ring breakage.	Activated oxygen concentration and target adsorption efficiency.	[49]
Catalyst regeneration	Reduced electron–hole complexation rate and active site recovery.	Catalyst stability and surface passivation inhibition strategies.	[49]

**Table 4:** Key influencing factors and optimization strategies for microbial degradation of PCDD/Fs.

Influencing Factors	Optimal Range/Conditions	Impact on Degradation Efficiency	Optimization Strategies	Ref.
Temperature	25–35 °C	1.5–2-fold increase in degradation rate for every 10 °C increase in temperature	Constant-temperature fermentation system	[161]
Oxygen concentration	Aerobic: DO > 2 mg/L	Aerobic degradation rates are 3–5 times higher than anaerobic	Aeration-enhanced or anaerobic membrane bioreactors	[148]
pH	Neutral (6.5–7.5)	Extreme pH (<5 or >9) inhibits enzyme activity	Buffer addition	[162]
Pollutant concentration	<100 μg/kg (soil)	High concentrations (>1 mg/kg) lead to inhibition of microbial toxicity	Biostimulation (addition of carbon sources)	[163]
Metabolite	Glucose, lignin	Co-metabolize to increase dechlorination efficiency by 20–40%	Exogenous carbon source replenishment	[164]

**Table 5:** Comprehensive comparison of four PCDD/F control technologies.

Technology Type	Removal Efficiency	Scalability	Cost	Environmental Risks	Ref.
Optimization technology of high-temperature pyrolysis and incineration	Removal efficiency over 99%, suitable for high-concentration PCDD/F waste	High scalability, suitable for large-scale industrial applications like municipal waste incineration	High equipment investment and operational costs	May generate secondary pollutants like NOx and SO_2_; requires strict exhaust gas treatment measures	[109,165]
Photocatalytic degradation technology	High removal efficiency for low-concentration PCDD/Fs, but less effective for high-concentration waste	Good scalability, suitable for small-scale pollution source treatment	Low cost, low energy consumption, but reliant on sunlight or artificial light sources	Potential catalyst photodegradation issues, minor water quality impacts	[126,143]
Supercritical water oxidation technology	High removal efficiency, removal rate up to 99%	Limited scalability, mainly applied in pilot or experimental stages	High equipment and technical requirements, limited commercial application	High-temperature, high-pressure conditions pose equipment risks; wastewater needs treatment	[119,166]
Biological detoxification technology	High removal efficiency for low-concentration PCDD/Fs, but slower treatment speed	Good scalability, suitable for soil, sediment, and solid waste remediation	Low cost, but long processing time, may require significant biological remediation resources	Environmentally friendly, but may require long-term treatment and has limitations on certain pollutants	[156,167]

## Data Availability

Data are contained within the article and Supplementary Materials.

## Supplementary Materials

The following supporting information can be downloaded at: https://www.mdpi.com/article/10.3390/molecules30204106/s1, PRISMA 2020 Checklist [183].
