# Supplementary material for: Methodological Insights into the Occurrence, Conversion, and Control of Polychlorinated Dibenzo-p-Dioxins/Dibenzofurans from Waste Incineration
Source: Molecules. 2025 Oct 16;30(20):4106. doi: 10.3390/molecules30204106 (PMC12566111; doi:10.3390/molecules30204106)
Supplement: Supplementary file 1 [file molecules-30-04106-s001.zip › molecules-3876849-supplementary.pdf]

PRISMA 2020 Checklist

Section and Topic	Item #	Checklist item	Location where item is reported
**TITLE**			
Title	1	Identify the report as a systematic review.	Not applicable.
**ABSTRACT**			
Abstract	2	See the PRISMA 2020 for Abstracts checklist.	Abstract section, page 2. The abstract summarizes the objective, methods, results, and conclusions.
**INTRODUCTION**			
Rationale	3	Describe the rationale for the review in the context of existing knowledge.	Introduction, page 3-8. Discusses the problem of PCDD/Fs from waste incineration and the need for effective control strategies.
Objectives	4	Provide an explicit statement of the objective(s) or question(s) the review addresses.	Introduction, final paragraph, page 7: " Considering the importance of hazardous substance reduction during waste incineration and the complexity of the related processes, in this review, we aim to systematically investigate the recent advances in methodologies for controlling the generation of PCDD/Fs and reducing their environmental pollution in three aspects: (1) generation mechanisms and transformation processes, (2) control technologies, and (3) challenges and perspectives."
**METHODS**			
Eligibility criteria	5	Specify the inclusion and exclusion criteria for the review and how studies were grouped for the syntheses.	Section 2.2. Eligibility Criteriat, page 10.
Information sources	6	Specify all databases, registers, websites, organisations, reference lists and other sources searched or consulted to identify studies. Specify the date when each source was last searched or consulted.	Section 2.1. Search Strategy, page 9: Databases (Web of Science, Scopus, PubMed) and date range (Jan 2000 - Dec 2024) are specified.
Search strategy	7	Present the full search strategies for all databases, registers and websites, including any filters and limits used.	Section 2.1. Search Strategy, page 9: Search terms and language limit are provided.
Selection process	8	Specify the methods used to decide whether a study met the inclusion criteria of the review, including how many reviewers screened each record and each report retrieved, whether they worked independently, and if applicable, details of automation tools used in the process.	Section 2.3. Study Selection, page 10: Describes the process of removing duplicates and screening titles/abstracts/full-texts. Indicate how many people are involved in each screening stage.
Data collection process	9	Specify the methods used to collect data from reports, including how many reviewers collected data from each report, whether they worked independently, any processes for obtaining or confirming data from study investigators, and if applicable, details of automation tools used in the process.	Not applicable.
Data items	10a	List and define all outcomes for which data were sought. Specify whether all results that were compatible with each outcome domain in each study were sought (e.g. for all measures, time points, analyses), and if not, the methods used to decide which results to collect.	The focus of the review is on mechanisms, technologies, and factors, rather than quantitative outcomes for meta-analysis. This is implicitly covered in the objectives and methods.
	10b	List and define all other variables for which data were sought (e.g. participant and intervention characteristics, funding sources). Describe any assumptions made about any missing or unclear information.	Not directly applicable in a standard way, as the "studies" are not clinical trials. The types of information extracted (e.g., incineration conditions, technology parameters, catalytic mechanisms) are described throughout the results.
Study risk of bias assessment	11	Specify the methods used to assess risk of bias in the included studies, including details of the tool(s) used, how many reviewers assessed each study and whether they worked independently, and if applicable, details of automation	Section 2.4. Quality Assessment, page 11.

Section and Topic	Item #	Checklist item	Location where item is reported
		tools used in the process.	
Effect measures	12	Specify for each outcome the effect measure(s) (e.g. risk ratio, mean difference) used in the synthesis or presentation of results.	Not applicable. This review is not a meta-analysis.
Synthesis methods	13a	Describe the processes used to decide which studies were eligible for each synthesis (e.g. tabulating the study intervention characteristics and comparing against the planned groups for each synthesis (item #5)).	The synthesis is structured by pre-defined themes (formation mechanisms, control technologies, etc.). This is described in the Introduction and Methods.
	13b	Describe any methods required to prepare the data for presentation or synthesis, such as handling of missing summary statistics, or data conversions.	Not applicable. No quantitative data synthesis was performed.
	13c	Describe any methods used to tabulate or visually display results of individual studies and syntheses.	The use of tables (Tables 1-5) and figures (Figs. 1-9) to summarize and display findings is evident throughout the manuscript.
	13d	Describe any methods used to synthesize results and provide a rationale for the choice(s). If meta-analysis was performed, describe the model(s), method(s) to identify the presence and extent of statistical heterogeneity, and software package(s) used.	The method of synthesis is narrative, based on thematic analysis and summary of findings from included studies. This is standard for this type of review.
	13e	Describe any methods used to explore possible causes of heterogeneity among study results (e.g. subgroup analysis, meta-regression).	Not applicable.
	13f	Describe any sensitivity analyses conducted to assess robustness of the synthesized results.	Not applicable.
Reporting bias assessment	14	Describe any methods used to assess risk of bias due to missing results in a synthesis (arising from reporting biases).	Not mentioned.
Certainty assessment	15	Describe any methods used to assess certainty (or confidence) in the body of evidence for an outcome.	Section 2.4. Quality Assessment, page 11. Reference is made to the use of GRADE to evaluate the quality of evidence.
**RESULTS**			
Study selection	16a	Describe the results of the search and selection process, from the number of records identified in the search to the number of studies included in the review, ideally using a flow diagram.	Section 2.3. Study Selection and Fig. 2 (PRISMA flow diagram), page 10-11.
	16b	Cite studies that might appear to meet the inclusion criteria, but which were excluded, and explain why they were excluded.	Section 2.3. Study Selection and Fig. 2 (PRISMA flow diagram), page 10-11.
Study characteristics	17	Cite each included study and present its characteristics.	This is fulfilled throughout the entire Results section (Sections 3, 4, 5), where findings from individual studies are cited and discussed. A summary table of included studies is not present but is not strictly necessary for this narrative format.
Risk of bias in studies	18	Present assessments of risk of bias for each included study.	Not applicable.
Results of individual studies	19	For all outcomes, present, for each study: (a) summary statistics for each group (where appropriate) and (b) an effect estimate and its precision (e.g. confidence/credible interval), ideally using structured tables or plots.	Not applicable. This is not a meta-analysis.
Results of syntheses	20a	For each synthesis, briefly summarise the characteristics and risk of bias among contributing studies.	The characteristics of contributing studies are integrated into the narrative. Risk of bias was not assessed per study.
	20b	Present results of all statistical syntheses conducted. If meta-analysis was done, present for each the summary estimate and its precision (e.g.	Not applicable.

PRISMA 2020 Checklist

Section and Topic	Item #	Checklist item	Location where item is reported
		confidence/credible interval) and measures of statistical heterogeneity. If comparing groups, describe the direction of the effect.	
	20c	Present results of all investigations of possible causes of heterogeneity among study results.	Not applicable.
	20d	Present results of all sensitivity analyses conducted to assess the robustness of the synthesized results.	Not applicable.
Reporting biases	21	Present assessments of risk of bias due to missing results (arising from reporting biases) for each synthesis assessed.	Not applicable.
Certainty of evidence	22	Present assessments of certainty (or confidence) in the body of evidence for each outcome assessed.	Not applicable.
**DISCUSSION**			
Discussion	23a	Provide a general interpretation of the results in the context of other evidence.	Section 5, page 56-66. Challenges and perspectives provides an interpretation and places the findings in the context of future needs. The discussion part summarizes the main findings related to the existing knowledge.
	23b	Discuss any limitations of the evidence included in the review.	Limitations are partially discussed in Section 5.1 (e.g., disparity between lab and real conditions), page 57.
	23c	Discuss any limitations of the review processes used.	Not explicitly discussed.
	23d	Discuss implications of the results for practice, policy, and future research.	Sections 5.2 (Industry standards) and 5.3 (National policy) cover policy implications. Section 5.1 and the conclusion of Section 5 cover future research directions.
**OTHER INFORMATION**			
Registration and protocol	24a	Provide registration information for the review, including register name and registration number, or state that the review was not registered.	The registration link of PROSPERO are mentioned in the 2. Method section, page 9.
	24b	Indicate where the review protocol can be accessed, or state that a protocol was not prepared.	No agreement prepared.
	24c	Describe and explain any amendments to information provided at registration or in the protocol.	There is no modification to the information provided at the time of registration or in the scheme.
Support	25	Describe sources of financial or non-financial support for the review, and the role of the funders or sponsors in the review.	Funding section, page 66.
Competing interests	26	Declare any competing interests of review authors.	Conflicts of Interest section, page 67.
Availability of data, code and other materials	27	Report which of the following are publicly available and where they can be found: template data collection forms; data extracted from included studies; data used for all analyses; analytic code; any other materials used in the review.	Not applicable.

From: Page MJ, McKenzie JE, Bossuyt PM, Boutron I, Hoffmann TC, Mulrow CD, et al. The PRISMA 2020 statement: an updated guideline for reporting systematic reviews. BMJ 2021;372:n71. doi: 10.1136/bmj.n71. This work is licensed under CC BY 4.0. To view a copy of this license, visit https://creativecommons.org/licenses/by/4.0/
